# Prevalence and clinical significance of unsuspected intracranial findings in patients undergoing oncological whole-body 18F-FDG PET/CT imaging

**DOI:** 10.3389/fnume.2025.1668088

**Published:** 2026-01-21

**Authors:** Diana Nyamieri, Samuel Nguku Gitau, Sheila Waa

**Affiliations:** 1 Radiology Department, Avenue Hospital, Nairobi, Kenya; 2 Radiology Department, Aga Khan University Hospital, Nairobi, Kenya

**Keywords:** 18F-FDG PET/CT, brain, metastasis, oncological, whole-body

## Abstract

**Purpose:**

Routine oncological whole-body 18F-1-fluoro-2-deoxyglucose (FDG) PET/CT in the majority of institutions is performed from the base of the skull to the mid-thigh. However, at our institution, the brain is included. The aim of this study was to identify the prevalence of unsuspected intracranial findings in patients undergoing oncological 18F-FDG PET/CT examinations with inclusion of the brain in the field of view.

**Methods:**

A total of 3,523 patients who underwent oncological limited whole-body 18F-FDG PET/CT scans between February 2019 and December 2021 were retrospectively reviewed. The study variables included the patient's age, sex, type of malignancy, disease stage, and clinical presentation and the presence of clinically unsuspected intracranial findings. The intracranial findings were correlated with brain MRI findings in a subset of patients. Clinical significance, defined by a change in disease stage and/or patient management informed by the identification of unsuspected intracranial findings, was assessed.

**Results:**

In total, 132/3,523 (3.7%) oncological whole-body 18F-FDG PET/CT scans had unsuspected intracranial findings, of which clinically significant unsuspected intracranial findings were found in 62 cases (1.4%). The most common intracranial findings were metastasis, followed by subclinical vascular findings. Moreover, 22/62 cases underwent follow-up brain MRI, and the sensitivity and specificity of the 18F-FDG PET/CT scans were 94.7% and 66.7%, respectively. Data on post-PET/CT management were available for 32/132 patients. A change in management was observed in 25/32 (78%) cases.

**Conclusion:**

The inclusion of the brain in the field of view in oncological whole-body 18F-FDG PET/CT may lead to the early detection of unsuspected intracranial metastases and changes in patient management. This is especially true for breast and lung cancers, which have a greater propensity to metastasize to the brain.

## Introduction

Cancer is one of the leading causes of death worldwide and resulted in approximately 10 million deaths in 2020 (1). In Kenya, cancer is among the top five leading causes of death despite attempts to improve early diagnosis and treatment (2, 3). The most common cancers in Kenya are breast, prostate, cervical, and colorectal cancers. It has been shown that detecting cancer early in the disease process is crucial for lowering mortality and economic burden and, therefore, prolonging survival (4).

Positron emission tomography/computed tomography (PET/CT) is commonly used for staging cancer and can demonstrate tumor function at the molecular level by using different radiotracers (5). The most used radiotracer is 18F-fluoro-2-deoxyglucose (18F-FDG), an analog of glucose (6). 18F-FDG PET/CT has a niche in the assessment of oncological cases and plays a vital role in not only staging and restaging but also the assessment of treatment response.

In recent years, 18F-FDG PET/CT has become a more fundamental investigation in many malignancies in patients undergoing diagnostic workups for treatment and management. There is no consensus regarding the field of view in patients undergoing whole-body (WB) PET/CT. The term “whole-body 18F-FDG PET/CT” is ambiguous as it frequently does not include the brain, portions of the skull, upper extremities, and lower extremities (7).

The Society of Nuclear Medicine and Molecular Imaging (SNMMI) describes three types of fields of view (FOV): whole-body, limited area tumor imaging, and limited whole-body. Although the SNMMI defines the limited whole-body FOV as from the skull base to the mid-thigh, five different limited FOVs have been categorized according to anatomical length, all of which have been described and utilized in practice (6, 8).

The majority of oncological 18F-FDG scans are acquired from the base of the skull to the mid-thigh and require between 6 and 18 min (acquisition time), depending on the generation of the PET/CT machine and the patient’s height (8). Moreover, when using newer total-body PET/CT scanners, acquisition time can be as low as 2 min if routine full 18F-FDG activity is administered (9). It has been postulated that there is little clinical benefit gained from adding the brain to the FOV since 18F-FDG PET/CT has low sensitivity for intracranial metastasis, especially those smaller than 1 cm, because the brain has intense physiological uptake of FDG that may mask lesions (9). The European Association of Nuclear Medicine (EANM) recommends extended whole-body examinations, including the brain, for tumors that show a high probability of intracranial metastases, such as lung and melanoma cancers (6). As much as the two societies have tried to summarize different fields of view, there is still ambiguity and variations in the definition of a limited whole-body field of view (10–12).

Intracranial metastases account for 15%–40% of patients with brain malignancies, many of whom do not present with any symptoms. There is a higher frequency of brain metastasis in certain primary malignancies such as melanoma, breast, gastrointestinal, ovary, cervix, and renal cancers (13). Computed tomography (CT) and magnetic resonance imaging (MRI) cross-sectional imaging are the most used modalities in the evaluation of brain metastasis. Brain MRI is the recommended imaging modality for the evaluation of metastasis (14).

Studies have shown that the routine addition of the brain in 18F-FDG PET examinations in oncological cases has occasionally identified previously unsuspected brain metastases (15). In a study of 500 whole-body PET/CT images (from the vertex to the feet), 8 (1.6%) patients had unsuspected brain metastases, with changes in disease staging in two patients based on the brain findings (10). The authors concluded that the addition of the brain and lower limbs in the field of view is useful as it leads to the identification of unsuspected metastatic sites. However, another study found that the prevalence of identifying previously unsuspected brain metastases on routine 18F-FDG PET/CT is low (0.7%) and few patients require a change in their stage of disease, recommending that oncological scans should include the skull base to the mid-thigh (9).

In view of this lack of consensus on the definition of the FOV and contrasting findings in previous studies, the aim of this study was to identify the prevalence of clinically unsuspected intracranial findings in patients who underwent routine oncological 18F-FDG PET/CT examinations with the inclusion of the whole brain in the field of view.

## Materials and methods

This was a retrospective, analytical, cross-sectional study involving a review of oncological 18F-FDG PET/CT examinations performed between February 2019 and December 2021 at Aga Khan University Hospital, Nairobi. Follow-up brain MRI performed within 6 weeks of the PET/CT was also assessed and correlated with the intracranial findings seen on PET/CT. As this was a retrospective study with no direct effect on the management of the patients involved in the study, a waiver of consent was sought and granted by the Institutional Scientific and Research Ethics Committee (RE: 2022/ISERC-12). All data were anonymized with all patient identifiers removed.

The study variables comprised the patient's type of malignancy, sex, age, and disease stage; the presence of unsuspected intracranial findings; and the clinical information provided. The clinical impact (change in disease stage or management) of the identified intracranial findings was also obtained from the available clinical notes.

Patients who underwent oncological 18F-FDG PET/CT imaging (vertex to mid-thigh or vertex to toes) during the study period were included. Patients with known or suspected intracranial findings based on clinical symptoms or previous imaging were excluded.

All the PET/CT scans were acquired after an uptake time of approximately 60 min following intravenous injection of 296–444 MBq (8–12 mCi) of 18F-FDG, with the patient having fasted for at least 4–6 h. All imaging was performed using a General Electric Discovery MI PET/CT scanner, which included 3D PET acquisition at 2.5 min per bed position and a low-dose CT for anatomical correlation and attenuation correction. All PET/CT images were reviewed by a nuclear medicine physician and consultant radiologist with over 5 years of experience.

All clinically unsuspected intracranial findings were documented. Metastases were distinguished from benign lesions on 18-FDG PET/CT by high FDG avidity above background brain uptake/contralateral brain area and the presence of perilesional edema on CT. In addition, hypometabolic foci on the PET images with corresponding abnormality on CT (a space-occupying lesion or vasogenic edema) were also considered metastatic.

The brain MRI examinations for the evaluation of the intracranial imaging findings were carried out on a 3Tesla Philips Ingenia magnet or 1.5 Tesla GE Signa Explorer scanner within 6 weeks of the antecedent 18F-FDG PET/CT and reviewed by experienced consultant radiologists.

Descriptive statistics were used to summarize the demographic and clinical characteristics of the study population using frequencies and percentages for categorical variables and mean (standard deviations) or median [interquartile range (IQR)] for continuous variables, depending on the distribution. Categorical variables measured at baseline (PET/CT) and during the follow-up MRI were compared descriptively.

## Results

### Baseline characteristics

**Figure 1:**
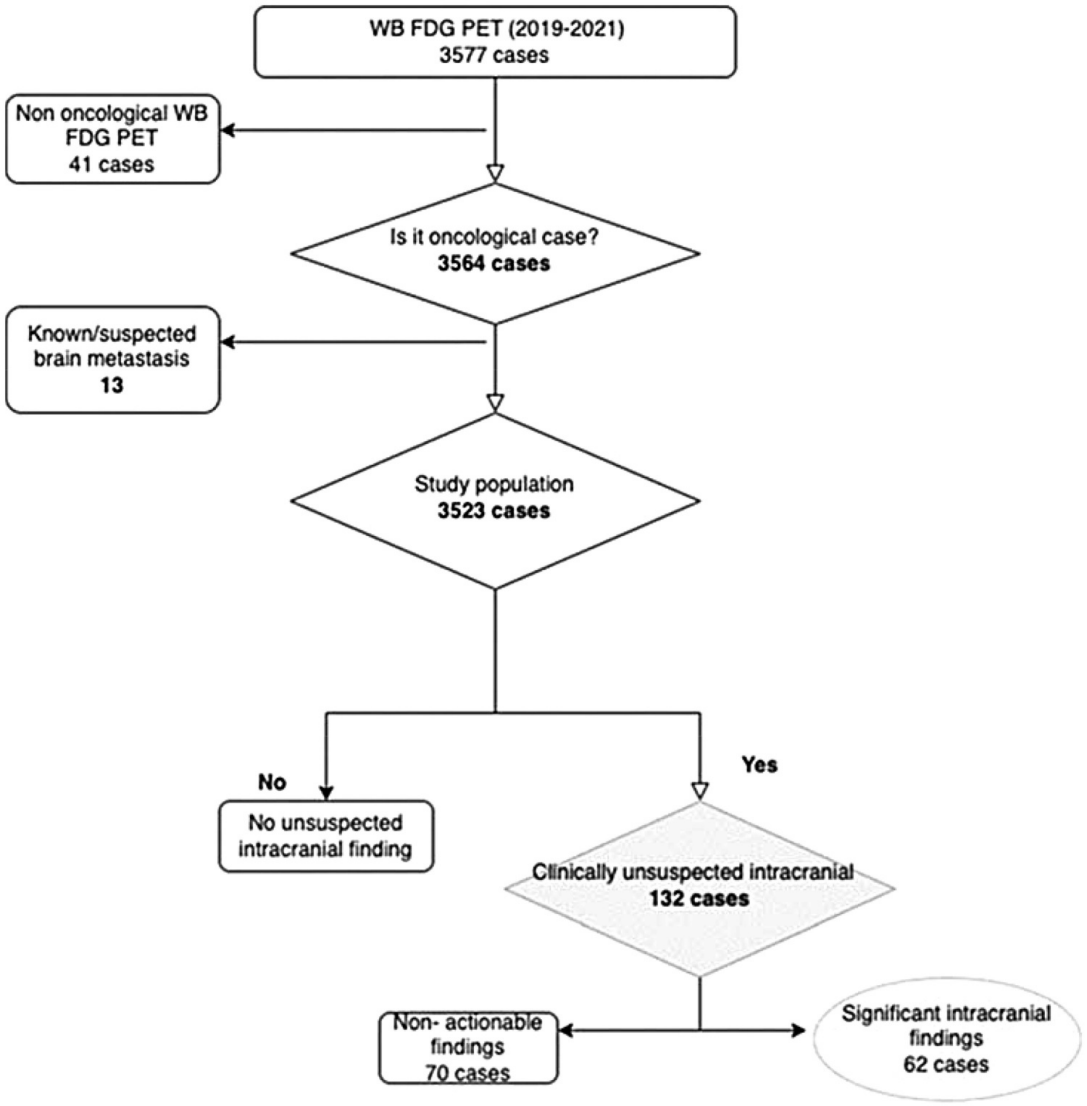
Schematic of the sample population.

A total of 3,577 whole-body 18F-FDG PET/CT scans were performed in the study period. Of these, 13 had known intracranial metastases and 41 cases were for non-oncological indications and were therefore excluded. Therefore, 3,523 cases were included in the analysis, as summarized in Figure 1.

The majority of the cases were female (2,215, 62.9%). The median age was 56 years (IQR 45–65).

The distribution of the most common malignancies was as follows: breast: 897 (25.4%); gastrointestinal: 861 (24%); hematological: 554 (16%); gynecological: 379 (11%); head and neck: 248 (7%); lung: 222 (6%); and genitourinary: 100 (3%). The demographic characteristics are presented in Table 1.

**Table 1:** Baseline characteristics of the included patients.

Characteristic	*N* = 3,523
Age in years, median (IQR)	56.0 (45.0–65.0)
Sex, *n* (%)	
Female	2,215 (62.9)
Male	1,308 (37.1)
Category of cancer, *n* (%)	
Breast	897 (25.4)
Endocrine tumor	39 (1.1)
Gastrointestinal	861 (24.4)
Genitourinary	100 (2.8)
Gynecological	379 (10.7)
Head and neck	248 (7)
Hematological	554 (15.7)
Lung	222 (6.3)
Other	223 (6.4)

### 18F-FDG PET/CT intracranial abnormalities

Clinically unsuspected brain lesions were detected in 132 cases (3.7%). Of these, 62 patients had significant intracranial findings (61 cases of metastasis and 1 case of a meningioma with significant brain edema). The most common clinically unsuspected intracranial finding was metastasis, accounting for 61/132 (46.2%) cases. The remainder of the findings were classified as subclinical vascular findings (60/132, 45.5%), including chronic white matter microangiopathy, subdural hematomas, encephalomalacia, and chronic infarcts, and benign lesions (11, 8.3%), such as arachnoid cysts, meningiomas, and pituitary macroadenomas.

**Figure 2:**
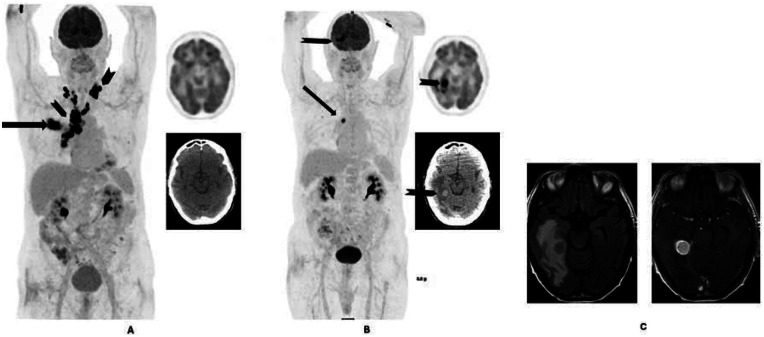
80-year-old male with lung cancer. The staging 18F-FDG PET/CT **(A)** from vertex to mid-thigh shows metabolically active right lung primary (arrow) with hilar, bilateral mediastinal, and supraclavicular nodal spread (arrowheads). Follow-up 18F-FDG PET/CT **(B)** 5 months after treatment with four cycles of carboplatin, premetrexed, and pembrolizumab shows a dramatic response in the primary disease sites and the nodes (arrow). However, there is a new focus of uptake in the brain (arrowhead) in keeping with brain metastasis. This was confirmed on MRI **(C)**, which shows an enhancing right temporal lobe lesion with perilesional edema. Brain metastasis was not clinically suspected at the time of the follow-up PET/CT.

The clinically significant unsuspected intracranial findings were metastases and one case of meningioma with significant surrounding edema (62/3,523, 1.4%). Breast and lung cancer accounted for the majority of the cases, with 24/61 (39.3%) and 9/61 (14.7%), respectively. The number of lung cancer, breast cancer, and melanoma cases that had metastasis to the brain in their individual cancer category was 9/222 (4%), 24/895 (2.6%), and 2/64 (3.1%), respectively. Figure 2 shows a patient with lung cancer who responded well to treatment but developed brain metastasis on follow up FDG PET CT which was confirmed on MRI brain.

Moreover, 47/61 (77%) cases of suspected intracranial metastasis had increased FDG uptake, while 14/61 (23%) cases showed reduced FDG uptake. The majority of the metastatic lesions were hypodense (57/61), and 4/61 were hyperdense, on non-contrast low-dose CT.

Of the 132 cases with intracranial findings, 22 had a follow-up MRI performed within 6 weeks. In 19/22 cases, there was no change in diagnosis between PET/CT and MRI findings. One case of suspected encephalomalacia on PET/CT was confirmed to be metastasis on the subsequent MRI and one case of suspected metastasis on PET/CT was confirmed as a chronic infarct on MRI. Another case of suspected metastasis on PET/CT was reported to be a glioblastoma on MRI (Table 2). The sensitivity and specificity of 18F-FDG PET/CT in identifying intracranial malignant lesions were 94.7% and 66.7%, respectively.

**Table 2:** PET/CT findings and MRI findings.

Primary cancer	Age	Sex	Intracranial finding on PET/CT	Brain MRI finding
Endocrine tumor	21	Female	Metastasis	Metastasis
Sarcoma	27	Male	Metastasis	Metastasis
Gynecological	34	Female	Metastasis	Metastasis
Breast	40	Female	Metastasis	Metastasis
Gynecological	40	Female	Metastasis	Metastasis
Breast	41	Female	Metastasis	Metastasis
Breast	45	Female	Encephalomalacia	Metastasis
Breast	46	Female	Metastasis	Metastasis
Breast	52	Female	Metastasis	Metastasis
Breast	54	Female	Metastasis	Metastasis
Primary cancer unknown	55	Female	Metastasis	Glioblastoma
Hematological	56	Female	Metastasis	Metastasis
Lung	59	Female	Metastasis	Metastasis
Breast	61	Female	Metastasis	Metastasis
Breast	61	Female	Metastasis	Metastasis
Breast	63	Female	Meningioma	Meningioma
Breast	63	Female	Metastasis	Metastasis
Head and neck	63	Male	Metastasis	Chronic infarct
Lung	64	Female	Metastasis	Metastasis
Breast	70	Female	Metastasis	Metastasis
Primary cancer unknown	71	Male	Pituitary macroadenoma	Pituitary macroadenoma
Lung	82	Male	Metastasis	Metastasis

### Change in management

**Figure 3:**
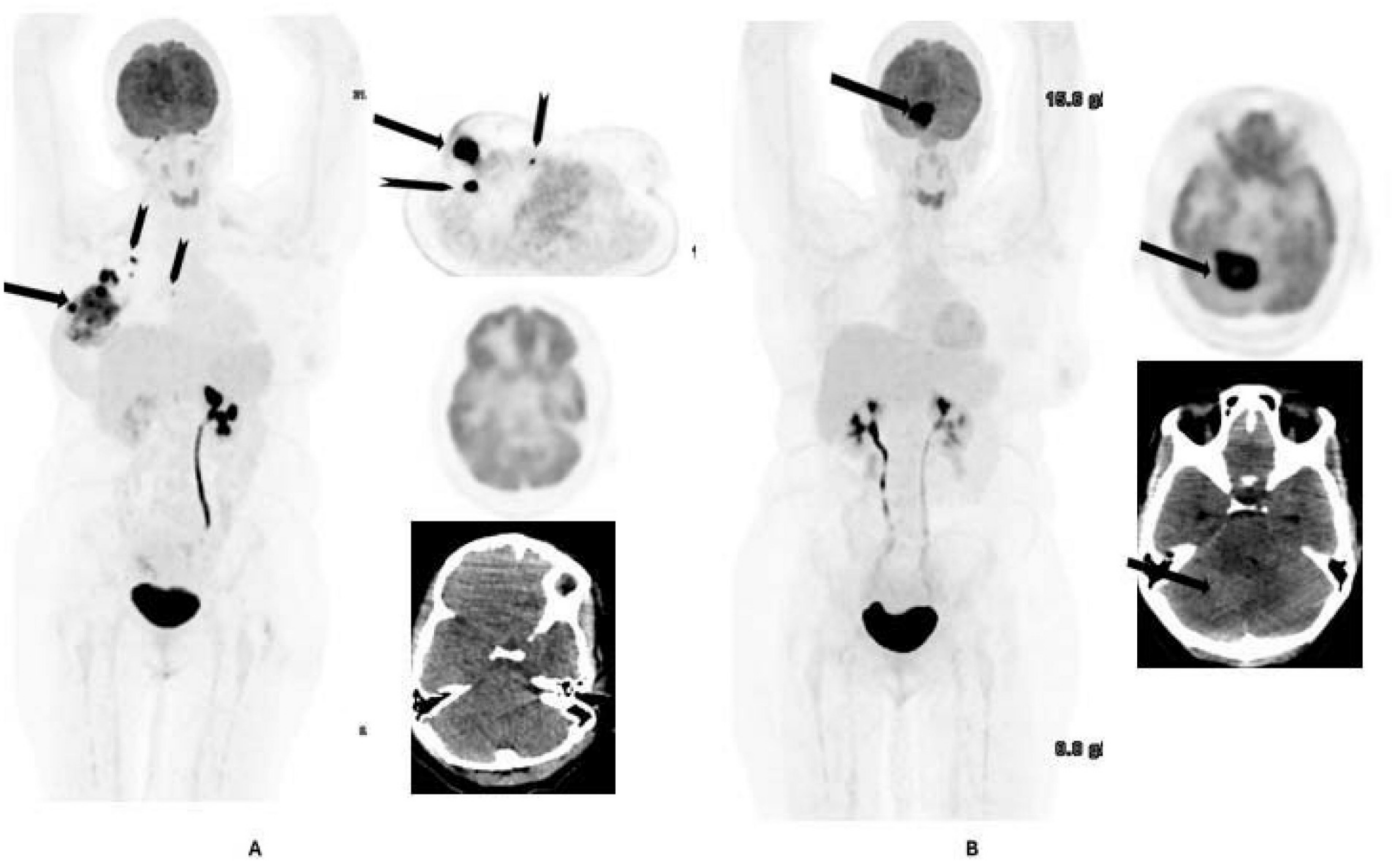
32-year-old female with right breast cancer. Baseline staging 18-F FDG PET/CT **(A)** shows metabolically active right primary breast cancer (arrow) with axillary and internal mammary nodal involvement (arrowheads). No brain lesion was identified at baseline. The patient was treated with neoadjuvant chemotherapy, mastectomy, and adjuvant radiotherapy. Follow-up 18F-FDG PET/CT **(B)** 6 months after completion of treatment shows an FDG-avid hyperdense right cerebellar lesion (arrows) suggestive of metastasis. No other site of FDG-avid disease was identified. Surgical excision of the lesion was conducted and revealed metastatic carcinoma consistent with primary breast cancer.

**Figure 4:**
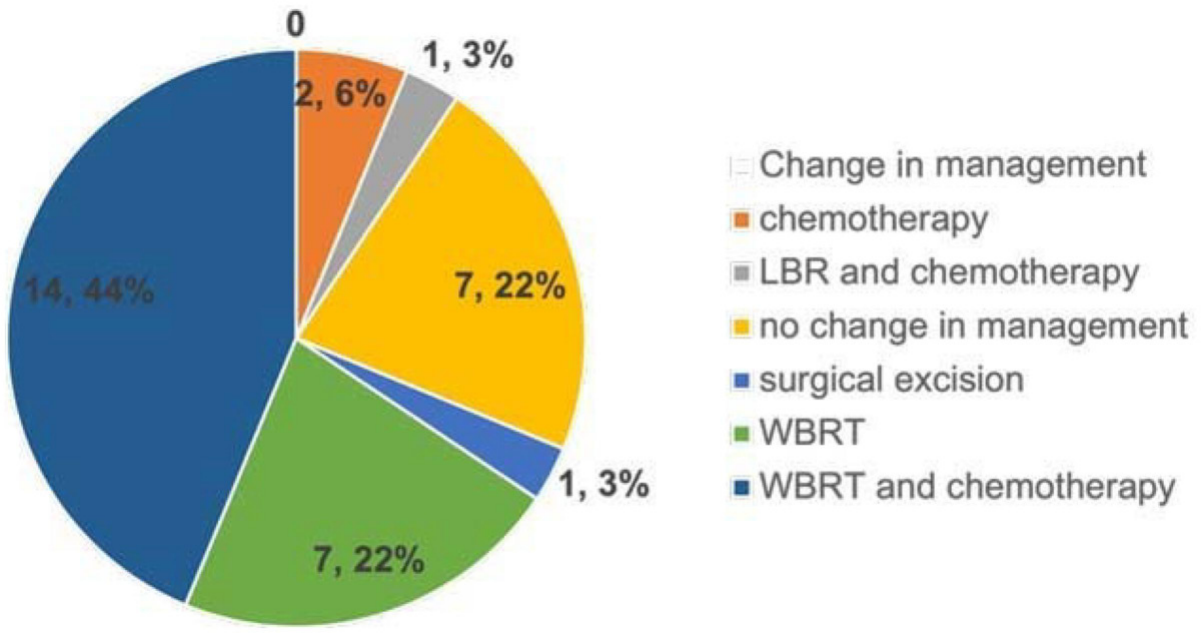
Pie chart showing the changes in management. LBR, localized brain radiotherapy; WBRT, whole-brain radiotherapy.

In total, 32 of the 132 cases with clinically unsuspected intracranial findings were followed up at our facility. Among these 32 cases, 25 required a change in management by undergoing brain radiation, chemotherapy, or surgery, while the remaining 7 cases required no change in management (Figure 4). The majority of those who required a change in management had metastatic lesions and one had a meningioma with surrounding edema, resulting in a significant mass effect. Two cases had changes in their cancer stage, with one having recurrence of breast cancer in the brain only (Figure 3) and the other from stage III to IV.

## Discussion

Whole-body 18F-FDG PET/CT with the inclusion of the brain in the field of view is not the standard protocol proposed by the EANM/SNMMI. The most commonly used protocol is from the base of the skull to the upper thigh (6). There are variations in the field of view used by individual institutions, which raises the question of whether the use of the standard proposed field of view for oncological WB PET/CT scans can result in an underestimation of the true extent of the disease. In our study, the inclusion of the brain in the field of view yielded significant intracranial findings that were clinically unsuspected beforehand. The prevalence of clinically unsuspected intracranial findings in our study was 3.8%, which is comparable to previous studies with prevalences of 2.1% and 3.1% (16, 17).

The prevalence of clinically significant unsuspected intracranial metastasis in our study was 1.4% and these cases had an impact on staging and management. Brain metastasis is the most common intracranial malignancy, accounting for 10%–40% (13, 18). Our findings indicate that there is a likelihood of brain metastases being asymptomatic and these may be missed in the initial staging and follow-up of patients. Additionally, PET/CT also identified a spectrum of intracranial findings, such as subclinical vascular lesions, benign lesions, subdural hematomas, chronic infarcts, and meningiomas. The appearance of a brain abnormality on PET/CT may yield relevant information for diagnosis, not only in metastasis but also in other intracranial findings, despite PET/CT having low spatial resolution (18).

In our study, breast cancer, lung cancer, and melanoma were among the most common cancers with unsuspected brain metastasis. This was similar to studies that show that melanoma and lung, breast, and renal cell cancers are the most common cancers that metastasize to the brain, which led to EANM proposing the inclusion of the brain in the FOV for lung cancer and melanoma (6). Among the cases with clinically unsuspected intracranial findings in our study that were followed up in our facility, 78% required a change in management and two patients not only required a change in management but also a change in cancer stage, which had an impact on these patients' outcomes. The majority of the changes in management were attributed to asymptomatic brain metastasis.

Admittedly, the sensitivity in detecting metastasis of 18F-FDG PET is low in comparison to brain MRI with contrast due to the better soft tissue contrast of the latter. However, a hybrid of 18F-FDG PET/CT has shown an added value in picking up brain metastasis and the mass effect caused by the lesions. In this study, a comparison of 18F-FDG PET/CT and brain MRI was conducted, which showed that 18F-FDG PET/CT had a sensitivity of 94% and a specificity of 66% in identifying brain metastases. The addition of intravenous contrast for the low-dose CT component further enhanced the sensitivity of 18F-FDG PET/CT, as the majority of the metastatic lesions showed enhancement. There was only one false negative case of suspected encephalomalacia/chronic infarct, which was later confirmed to be a metastatic lesion on brain MRI. In this case, the lesion was small and resulted in vasogenic edema without increased FDG uptake. This highlights the limitation of both 18F-FDG PET and low-dose CT in identifying small brain metastases presenting as hypometabolic areas secondary to vasogenic edema. There were two false positive cases. One was a chronic infarct, which again highlights the challenge with hypometabolic lesions in the brain as discussed. The other was a primary brain neoplasm (glioblastoma), which can be difficult to distinguish from metastasis on brain 18F-FDG PET/CT. The results in this study were comparable to a previous study that showed a sensitivity of 75% (7).

The majority of the clinically unsuspected metastatic lesions were seen on the 18F-FDG PET component as areas of increased uptake and had an anatomical correlation with the low-dose CT component. The combination of 18F-FDG PET and CT increases the sensitivity of PET/CT even though low-dose CT has poor sensitivity. In total, 20% of the metastatic lesions showed low FDG uptake, which can be attributed to the surrounding edema and has been described previously (19).

It can be argued that the inclusion of the brain in the field of view increases the radiation dose to the patient; however, in our study, the majority of the cases comprised low-dose CT scans for anatomical correlation and these may not have significantly increased the effective dose to the patients. The effective radiation dose contributed by low-dose CT to the patient's overall radiation dose during 18F-FDG PET/CT examination is 20% of the total radiation dose and close to a negligible percentage is contributed by brain and neck CT (20). The effective dose associated with the CT exam (EDCT) is calculated from the dose length product. The effective dose per unit dose-length product varies for different parts of the body. For the head, it is 0.0021 mSv/mGy-cm, which translates to very minimal additional dose to the patient for head CT image acquisition (21). The newer generation PET/CT scanners have a short acquisition time (1–2 min per bed position) for PET. Furthermore, with the recent development of total-body PET/CT scanners with a larger FOV, acquisition time for the whole body can be as short as 2 min if routine full FDG activity is administered (22). Therefore, including the brain in the routine whole-body oncological 18F-FDG PET/CT has the potential benefit of identifying unsuspected intracranial findings without significantly increasing the radiation dose to the patient or the acquisition time.

Our study had a few limitations. First, not all the intracranial abnormalities were confirmed with a biopsy, and therefore, there is a potential for overestimating the true positives in the study. Additionally, this study was retrospective with missing data for some patients, especially a lack of MRI correlation for all patients. We were, therefore, unable to determine the diagnostic accuracy of 18F-FDG PET/CT in all our cases since not all the patients underwent MRI brain examinations. However, our aim was not to determine the sensitivity and specificity of 18F-FDG PET/CT but rather to show the potential added value of the inclusion of the brain in the field of view. A larger prospective multi-institutional study to determine the sensitivity and specificity of 18F-FDG PET/CT using brain MRI as the reference test would be beneficial.

## Conclusion

The prevalence of clinically unsuspected intracranial findings in patients undergoing oncological 18F-FDG PET/CT at our institution was 3.8%, with 1.4% of the cases resulting in changes in stage and management. These findings support the value of including the brain in the field of view in oncological 18F-FDG PET/CT, especially in breast and lung cancers, which have a greater propensity to metastasize to the brain. This could potentially reduce the unfavorable outcomes that result from a delayed diagnosis of brain metastasis, especially in resource-poor settings where 18F-FDG PET/CT may be the only staging examination performed on a patient.

## Data Availability

The raw data supporting the conclusions of this article will be made available by the authors, without undue reservation.
